# Prevention and Control of Seasonal Influenza with Vaccines: Recommendations of the Advisory Committee on Immunization Practices—United States, 2018–19 Influenza Season

**DOI:** 10.15585/mmwr.rr6703a1

**Published:** 2018-08-24

**Authors:** Lisa A. Grohskopf, Leslie Z. Sokolow, Karen R. Broder, Emmanuel B. Walter, Alicia M. Fry, Daniel B. Jernigan

**Affiliations:** 1 Influenza Division, National Center for Immunization and Respiratory Diseases, CDC; 2 Battelle Memorial Institute, Atlanta, Georgia; 3 Immunization Safety Office, National Center for Emerging and Zoonotic Infectious Diseases, CDC; 4 Duke University School of Medicine, Durham, North Carolina

## Abstract

This report updates the 2017–18 recommendations of the Advisory Committee on Immunization Practices (ACIP) regarding the use of seasonal influenza vaccines in the United States (MMWR Recomm Rep 2017;66[No. RR-2]). Routine annual influenza vaccination is recommended for all persons aged ≥6 months who do not have contraindications. A licensed, recommended, and age-appropriate vaccine should be used. Inactivated influenza vaccines (IIVs), recombinant influenza vaccine (RIV), and live attenuated influenza vaccine (LAIV) are expected to be available for the 2018–19 season. Standard-dose, unadjuvanted, inactivated influenza vaccines will be available in quadrivalent (IIV4) and trivalent (IIV3) formulations. Recombinant influenza vaccine (RIV4) and live attenuated influenza vaccine (LAIV4) will be available in quadrivalent formulations. High-dose inactivated influenza vaccine (HD-IIV3) and adjuvanted inactivated influenza vaccine (aIIV3) will be available in trivalent formulations.

Updates to the recommendations described in this report reflect discussions during public meetings of ACIP held on October 25, 2017; February 21, 2018; and June 20, 2018. New and updated information in this report includes the following four items. First, vaccine viruses included in the 2018–19 U.S. trivalent influenza vaccines will be an A/Michigan/45/2015 (H1N1)pdm09–like virus, an A/Singapore/INFIMH-16-0019/2016 (H3N2)-like virus, and a B/Colorado/06/2017–like virus (Victoria lineage). Quadrivalent influenza vaccines will contain these three viruses and an additional influenza B vaccine virus, a B/Phuket/3073/2013–like virus (Yamagata lineage). Second, recommendations for the use of LAIV4 (FluMist Quadrivalent) have been updated. Following two seasons (2016–17 and 2017–18) during which ACIP recommended that LAIV4 not be used, for the 2018–19 season, vaccination providers may choose to administer any licensed, age-appropriate influenza vaccine (IIV, RIV4, or LAIV4). LAIV4 is an option for those for whom it is appropriate. Third, persons with a history of egg allergy of any severity may receive any licensed, recommended, and age-appropriate influenza vaccine (IIV, RIV4, or LAIV4). Additional recommendations concerning vaccination of egg-allergic persons are discussed. Finally, information on recent licensures and labeling changes is discussed, including expansion of the age indication for Afluria Quadrivalent (IIV4) from ≥18 years to ≥5 years and expansion of the age indication for Fluarix Quadrivalent (IIV4), previously licensed for ≥3 years, to ≥6 months.

This report focuses on the recommendations for use of vaccines for the prevention and control of influenza during the 2018–19 season in the United States. A Background Document containing further information and a brief summary of these recommendations are available at https://www.cdc.gov/vaccines/hcp/acip-recs/vacc-specific/flu.html.

These recommendations apply to U.S.-licensed influenza vaccines used within Food and Drug Administration–licensed indications. Updates and other information are available at CDC’s influenza website (https://www.cdc.gov/flu). Vaccination and health care providers should check CDC’s influenza website periodically for additional information.

## Introduction

Influenza viruses typically circulate in the United States annually, most commonly from late fall through early spring. Most persons who contract influenza will recover without sequelae. However, influenza can cause serious illness, hospitalization, and death, particularly among older adults, very young children, pregnant women, and those with certain chronic medical conditions (*1*–*6*).

Routine annual influenza vaccination for all persons aged ≥6 months who do not have contraindications has been recommended by CDC and CDC’s Advisory Committee on Immunization Practices (ACIP) since 2010 (*7*). This report updates the 2017–18 ACIP recommendations regarding the use of seasonal influenza vaccines (*8*) and provides recommendations and guidance for vaccine providers regarding the use of influenza vaccines for the 2018–19 season. A variety of different formulations of influenza vaccine are available (Table 1). Contraindications and precautions to the use of influenza vaccines are summarized (Table 2). Abbreviations are used in this report to denote the various types of vaccines (Box).

**TABLE 1:** Influenza vaccines — United States, 2018–19 influenza season*

Trade name (Manufacturer)	Presentation	Age indication	HA (IIVs and RIV4) or virus count (LAIV4) per dose (each vaccine virus)	Egg-grown virus,^†^ cell culture-grown virus, or recombinant HA	Adjuvanted (Yes/No)	Latex (Yes/No)	Route	Thimerosal (Yes/No) If Yes, mercury *µ*g/0.5mL
**Quadrivalent IIVs (IIV4s)—Standard Dose—Contain inactivated virus**								
Afluria Quadrivalent (Seqirus)	0.5 mL PFS	≥5 yrs	15 *µ*g/0.5 mL	Egg	No	No	IM^§^	No
		≥5 yrs (needle/syringe)						Yes (24.5)
	5.0 mL MDV	18 through 64 yrs (jet injector)						
Fluarix Quadrivalent (GlaxoSmithKline)	0.5 mL PFS	≥6 mos	15 *µ*g/0.5 mL	Egg	No	No	IM§	No
Flulaval Quadrivalent (ID Biomedical Corp. of Quebec)	0.5 mL PFS	≥6 mos	15 *µ*g/0.5 mL	Egg	No	No	IM^§^	No
	5.0 mL MDV							Yes (<25)
Fluzone Quadrivalent (Sanofi Pasteur)	0.25 mL PFS	6 through 35 mos	7.5 *µ*g/0.25 mL 15 *µ*g/0.5 mL	Egg	No	No	IM^§^	No
	0.5 mL PFS	≥3 yrs						No
	0.5 mL SDV	≥3 yrs						No
	5.0 mL MDV	≥6 mos						Yes (25)
Flucelvax Quadrivalent (Seqirus)	0.5 mL PFS	≥4 yrs	15 *µ*g/0.5 mL	Cell culture	No	No	IM^§^	No
	5.0 mL MDV							Yes (25)
**Trivalent IIV (IIV3)—Standard Dose—Contains inactivated virus**								
Afluria (Seqirus)	0.5 mL PFS	≥5 yrs	15 *µ*g/0.5 mL	Egg	No	No	IM^§^	No
	5.0 mL MFV	≥5 yrs (needle/syringe)						Yes (24.5)
		18 through 64 yrs (jet injector)						
**Trivalent IIV3—High-Dose—Contains inactivated virus**								
Fluzone High-Dose (Sanofi Pasteur)	0.5 mL PFS	≥65 yrs	60 *µ*g/0.5 mL	Egg	No	No	IM^§^	No
**Trivalent IIV3—Adjuvanted—Contains inactivated virus**								
Fluad (Seqirus)	0.5 mL PFS	≥65 yrs	15 *µ*g/0.5 mL	Egg	Yes (MF59)	No	IM^§^	No
**Quadrivalent RIV (RIV4)—Contains recombinant HA**								
Flublok Quadrivalent (Sanofi Pasteur)	0.5 mL PFS	≥18 yrs	45 *µ*g/0.5 mL	Recombinant	No	No	IM^§^	No
**Quadrivalent LAIV (LAIV4)—Contains live, attenuated, cold-adapted virus**								
FluMist Quadrivalent (AstraZeneca)	0.2 mL prefilled single-use intranasal sprayer	2 through 49 yrs	10^6.5–7.5^ fluorescent focus units/0.2 mL	Egg	No	No	NAS	No

**Abbreviations:** ACIP = Advisory Committee on Immunization Practices; HA = hemagglutinin; IIV = inactivated influenza vaccine; IM = intramuscular; LAIV = live attenuated influenza vaccine; MDV = multidose vial; NAS = intranasal; PFS = prefilled syringe; RIV=recombinant influenza vaccine; SDV = single-dose vial.

* Immunization providers should consult Food and Drug Administration–approved prescribing information for 2018–19 influenza vaccines for the most complete and updated information, including (but not limited to) indications, contraindications, warnings, and precautions. Package inserts for U.S.-licensed vaccines are available at https://www.fda.gov/BiologicsBloodVaccines/Vaccines/ApprovedProducts/ucm093833.htm. Availability of specific products and presentations might change and differ from what is described in this table and in the text of this report.

^†^ Persons with a history of egg allergy may receive any licensed, recommended, age-appropriate influenza vaccine (IIV, RIV4, or LAIV4) that is otherwise appropriate for their health status. Those who report having had reactions to egg involving symptoms other than urticaria (hives), such as angioedema, respiratory distress, lightheadedness, or recurrent emesis; or who required epinephrine or another emergency medical intervention, should be vaccinated in an inpatient or outpatient medical setting (including, but not necessarily limited to, hospitals, clinics, health departments, and physician offices). Vaccine administration should be supervised by a health care provider who is able to recognize and manage severe allergic conditions.

^§^ For adults and older children, the recommended site for intramuscular influenza vaccination is the deltoid muscle. The preferred site for infants and young children is the anterolateral aspect of the thigh. Specific guidance regarding site and needle length for intramuscular administration is available in the ACIP General Best Practice Guidelines for Immunization, available at https://www.cdc.gov/vaccines/hcp/acip-recs/general-recs/index.html.

**TABLE 2:** Contraindications and precautions to the use of influenza vaccines — United States, 2018–19 influenza season*

Vaccine type	Contraindications	Precautions
IIV	History of severe allergic reaction to any component of the vaccine^†^ or after a previous dose of any influenza vaccine	Moderate or severe acute illness with or without fever
		History of Guillain-Barré syndrome within 6 weeks of receipt of influenza vaccine
RIV	History of severe allergic reaction to any component of the vaccine	Moderate or severe acute illness with or without fever
		History of Guillain-Barré syndrome within 6 weeks of receipt of influenza vaccine
LAIV	History of severe allergic reaction to any component of the vaccine^†^ or after a previous dose of any influenza vaccine	Moderate or severe acute illness with or without fever
	Concomitant aspirin- or salicylate-containing therapy in children and adolescents	History of Guillain-Barré syndrome within 6 weeks of receipt of influenza vaccine
	Children aged 2 through 4 years who have received a diagnosis of asthma or whose parents or caregivers report that a health care provider has told them during the preceding 12 months that their child had wheezing or asthma or whose medical record indicates a wheezing episode has occurred during the preceding 12 months	Asthma in persons aged ≥5 years Other underlying medical conditions that might predispose to complications after wild-type influenza infection (e.g., chronic pulmonary, cardiovascular [except isolated hypertension], renal, hepatic, neurologic, hematologic, or metabolic disorders [including diabetes mellitus])
	Children and adults who are immunocompromised due to any cause (including immunosuppression caused by medications or by HIV infection)	
	Close contacts and caregivers of severely immunosuppressed persons who require a protected environment	
	Pregnancy	
	Receipt of influenza antiviral medication within the previous 48 hours	

**Abbreviations:** ACIP = Advisory Committee on Immunization Practices; IIV = Inactivated Influenza Vaccine; LAIV = Live-Attenuated Influenza Vaccine; RIV = Recombinant Influenza Vaccine.

* Immunization providers should check Food and Drug Administration–approved prescribing information for 2018–19 influenza vaccines for the most complete and updated information, including (but not limited to) indications, contraindications, and precautions. Package inserts for US-licensed vaccines are available at https://www.fda.gov/BiologicsBloodVaccines/Vaccines/ApprovedProducts/ucm093833.htm.

^†^ History of severe allergic reaction (e.g., anaphylaxis) to egg is a labeled contraindication to the use of IIV and LAIV. However, ACIP recommends that any licensed, recommended, and age-appropriate influenza vaccine (IIV, RIV, or LAIV) may be administered to persons with egg allergy of any severity (see Persons with a History of Egg Allergy for further recommendations and information).

BOX **Abbreviation conventions for vaccines discussed in this report**

- IIV = Inactivated Influenza Vaccine
- RIV = Recombinant Influenza Vaccine
- LAIV = Live Attenuated Influenza Vaccine
- Numerals following letter abbreviations indicate valence
  - 3 for trivalent vaccines
  - 4 for quadrivalent vaccines
- Prefixes are used when necessary to refer to some specific vaccine types
  - a for adjuvanted vaccine (e.g., aIIV3)
  - cc for cell culture-based vaccine (e.g., ccIIV4)
  - HD for high-dose vaccine (e.g., HD-IIV3)
  - SD for standard-dose vaccine (e.g., SD-IIV3)

This report focuses on the recommendations for use of influenza vaccines for the prevention and control of influenza during the 2018–19 season in the United States. A summary of these recommendations and a Background Document containing additional information on influenza-associated illnesses and influenza vaccines are available at https://www.cdc.gov/vaccines/hcp/acip-recs/vacc-specific/flu.html.

## Methods

ACIP provides annual recommendations for the use of influenza vaccines for the prevention and control of influenza. The ACIP Influenza Work Group meets by teleconference once to twice per month throughout the year. Work Group membership includes several voting members of ACIP and representatives of ACIP Liaison Organizations.^*^ Discussions include topics such as influenza surveillance, vaccine effectiveness and safety, vaccine coverage, program feasibility, cost-effectiveness, and vaccine supply. Presentations are requested from invited experts, and published and unpublished data are discussed.

In general, the Background Document is updated to reflect recent additions to the literature related to the following: 1) recommendations that were made in previous seasons, 2) changes in the viral antigen composition of seasonal influenza vaccines, and 3) minor changes in guidance for the use of influenza vaccines (e.g., guidance for timing of vaccination and other programmatic issues, guidance for dosage in specific populations, guidance for selection of vaccines for specific populations that are already recommended for vaccination, and changes that reflect use that is consistent with Food and Drug Administration [FDA]–licensed indications and prescribing information). The summary included in the Background Document for such topics is not a systematic review but is intended to provide a broad overview of current literature. In general, systematic review and evaluation of the evidence using the Grading of Recommendations Assessment, Development and Evaluation (GRADE) approach is performed for new recommendations or substantial changes in the recommendations (e.g., expansion of the recommendation for influenza vaccination to new populations not previously recommended for vaccination or potential preferential recommendations for specific vaccines).

Updates and changes to the recommendations described in this report are of four types: 1) the vaccine virus composition for 2018–19 U.S. seasonal influenza vaccines; 2) a recommendation for the 2018–19 season that LAIV4 is an option for influenza vaccination of persons for whom it is appropriate; 3) a recommendation that persons with a history of egg allergy may receive any licensed, recommended, and age-appropriate influenza vaccine (IIV, RIV4, or LAIV4; and 4) recent regulatory actions, including new vaccine licensures and labeling changes for previously licensed vaccines. Information relevant to these changes included the following:

1. Recommendations for the composition of Northern Hemisphere influenza vaccines are made by the World Health Organization (WHO), which organizes a consultation, generally in February of each year. Surveillance data are reviewed and candidate vaccine viruses are discussed. A summary of the WHO meeting for selection of the 2018–19 Northern Hemisphere vaccine viruses is available at http://www.who.int/influenza/vaccines/virus/recommendations/201802_recommendation.pdf. Subsequently, FDA, which has regulatory authority over vaccines in the United States, convenes a meeting of its Vaccines and Related Biological Products Advisory Committee (VRBPAC). This committee considers the recommendations of WHO, reviews and discusses similar data, and makes a final decision regarding vaccine virus composition for influenza vaccines licensed and marketed in the United States. A summary of the FDA VRBPAC meeting of March 1, 2018, at which the composition of the 2018–19 U.S. influenza vaccines was discussed, is available at https://www.fda.gov/downloads/AdvisoryCommittees/CommitteesMeetingMaterials/BloodVaccinesandOtherBiologics/VaccinesandRelatedBiologicalProductsAdvisoryCommittee/UCM602610.pdf.
2. Regarding the recommendation for the 2018–19 season that LAIV4 is an option for influenza vaccination of those for whom it is appropriate, ACIP reviewed data from three sources in February 2018. Two were evaluations of previous seasons’ data concerning LAIV effectiveness among children aged 2 through 17 years, including 1) a combined individual-patient-level analysis of data from five U.S. observational studies of LAIV effectiveness for the 2013–14 through 2015–16 seasons, and 2) a systematic review and meta-analysis of U.S. and non-U.S. observational studies of LAIV effectiveness for the 2010–11 through 2016–17 seasons (for which further details are available in the Appendix to the Background Document). These observational data indicated that LAIV was poorly effective against influenza A(H1N1)pdm09-like viruses, and was significantly less effective than IIV against these viruses. However, LAIV was effective against influenza B viruses, and effectiveness of LAIV and IIV against influenza A(H3N2) viruses generally did not differ significantly. No estimates of the effectiveness of the current formulation of LAIV4, which contains a new H1N1pdm09-like vaccine virus, were available at the time of this review. The third source was manufacturer data concerning shedding and immunogenicity associated with administration of LAIV containing the new H1N1pdm09-like vaccine virus, A/Slovenia/2903/2015, among children aged 24 months through <4 years. These data suggest that this new H1N1pdm09-like virus has improved replicative fitness over previous H1N1pdm09-like viruses included in LAIV.
3. Regarding recommendations for persons with a history of egg allergy, persons with egg allergy of any severity were previously recommended to receive any licensed, recommended, and age-appropriate influenza vaccine. Use of LAIV4 for persons with egg allergy of any severity was approved by ACIP in February 2016, prior to the recommendations made for the 2016–17 and 2017–18 seasons that LAIV4 not be used in any population because of effectiveness concerns (*9*). In discussing LAIV4 for egg-allergic persons in February 2016, ACIP heard data from three studies which evaluated the use of LAIV in egg-allergic children, in which no cases of anaphylaxis occurred (*10*–*12*).
4. With regard to recommendations for newly licensed influenza vaccines and changes to the licensed indications for existing vaccines, ACIP relies on FDA for review of safety, immunogenicity, and effectiveness data pertaining to the licensure of influenza vaccines. Regulatory information pertinent to the labeling change for Afluria Quadrivalent (IIV4) is available at https://www.fda.gov/BiologicsBloodVaccines/Vaccines/ApprovedProducts/ucm518291.htm. Regulatory information pertinent to the labeling change for Fluarix Quadrivalent (IIV4) is available at https://www.fda.gov/BiologicsBloodVaccines/Vaccines/ApprovedProducts/ucm342391.htm.

## Primary Changes and Updates in the Recommendations

Routine annual influenza vaccination of all persons aged ≥6 months without contraindications continues to be recommended. No preferential recommendation is made for one influenza vaccine product over another for persons for whom more than one licensed, recommended, and appropriate product is available. Updated information and guidance in this report includes the following:

- Vaccine viruses included in the 2018–19 U.S. trivalent influenza vaccines will be an A/Michigan/45/2015 (H1N1)pdm09–like virus, an A/Singapore/INFIMH-16-0019/2016 (H3N2)-like virus, and a B/Colorado/06/2017–like virus (Victoria lineage). Quadrivalent influenza vaccines will contain these three viruses and an additional influenza B vaccine virus, a B/Phuket/3073/2013–like virus (Yamagata lineage).
- Following two seasons (2016–17 and 2017–18) during which ACIP recommended that LAIV4 not be used, ACIP voted in February 2018 to recommend that for the 2018–19 season, vaccination providers may choose to administer any licensed, age-appropriate influenza vaccine (IIV, RIV4, or LAIV4). LAIV4 is an option for those for whom it is appropriate (Table 2).
- Persons with a history of egg allergy of any severity may receive any licensed, recommended, and age-appropriate influenza vaccine (IIV, RIV4, or LAIV4). IIV and RIV4 have been previously recommended. Use of LAIV4 for persons with egg allergy was approved by ACIP in February 2016. Additional recommendations concerning vaccination of egg-allergic persons are discussed.
- Two recent regulatory actions are described. In August 2017, FDA approved an expanded age indication for Afluria Quadrivalent (IIV4). Previously licensed for ages ≥18 years, Afluria Quadrivalent is now licensed for ages ≥5 years. In January 2018, FDA approved an expanded age indication for Fluarix Quadrivalent (IIV4). Previously licensed for persons aged ≥3 years, Fluarix Quadrivalent is now licensed for persons aged ≥6 months. Children aged 6 through 35 months may receive Fluarix Quadrivalent at the same 0.5 mL per dose (containing 15 *µ*g of hemagglutinin [HA] per vaccine virus) as is used for older children and adults. This licensure creates a third option for inactivated influenza vaccines for children aged 6 through 35 months, in addition to the previously available 0.5 mL per dose (containing 15 *µ*g of HA per vaccine virus) presentation of FluLaval Quadrivalent (IIV4) and 0.25 mL per dose presentation (containing 7.5 *µ*g of HA per vaccine virus) of Fluzone Quadrivalent (IIV4).

## Recommendations for the Use of Influenza Vaccines, 2018–19

### Groups Recommended for Vaccination

Routine annual influenza vaccination is recommended for all persons aged ≥6 months who do not have contraindications. Recommendations regarding timing of vaccination, considerations for specific populations, the use of specific vaccines, and contraindications and precautions are summarized in the sections that follow.

### Timing of Vaccination

Balancing considerations regarding the unpredictability of timing of onset of the influenza season and concerns that vaccine-induced immunity might wane over the course of a season, it is recommended that vaccination should be offered by the end of October. Children aged 6 months through 8 years who require 2 doses (see Children Aged 6 Months Through 8 Years) should receive their first dose as soon as possible after vaccine becomes available, to allow the second dose (which must be administered ≥4 weeks later) to be received by the end of October. Community vaccination programs should balance maximizing likelihood of persistence of vaccine-induced protection through the season with avoiding missed opportunities to vaccinate or vaccinating after onset of influenza circulation occurs. Revaccination later in the season of persons who have already been fully vaccinated is not recommended. Vaccination should continue to be offered as long as influenza viruses are circulating and unexpired vaccine is available. To avoid missed opportunities for vaccination, providers should offer vaccination during routine health care visits and hospitalizations.

Optimally, vaccination should occur before onset of influenza activity in the community. However, because timing of the onset, peak, and decline of influenza activity varies, the ideal time to start vaccinating cannot be predicted each season. Moreover, more than one outbreak might occur in a given community in a single year. In the United States, localized outbreaks that indicate the start of seasonal influenza activity can occur as early as October. However, in 75% of influenza seasons from 1982–83 through 2017–18, peak influenza activity (which often is close to the midpoint of influenza activity for the season) has not occurred until January or later, and in 58% of seasons, the peak was in February or later (*13*).

A number of observational studies (*14*–*21*) and a post hoc analysis from a randomized controlled trial (*22*) have reported decreases in vaccine effectiveness (VE) within a single influenza season, with increasing time postvaccination. Waning effects have not been observed consistently across age groups, virus subtypes, and seasons; and observed declines in protection could be at least in part attributable to bias, unmeasured confounding, or the late season emergence of antigenic drift variants that are less well-matched to the vaccine strain. Some studies suggest this occurs to a greater degree with A(H3N2) viruses than with A(H1N1) or B viruses (*19*,*21*). This effect might also vary with recipient age; in some studies waning was more pronounced among older adults (*14*,*15*) and younger children (*15*). Rates of decline in VE have also varied. A multiseason (2011–12 through 2014–15) analysis from the U.S. Influenza Vaccine Effectiveness (U.S. Flu VE) Network found that VE declined by about 7% per month for H3N2 and influenza B, and 6%–11% per month for H1N1pdm09 (*16*). VE remained greater than zero for at least 5 to 6 months after vaccination. An analysis from the 2011–12 through 2013–14 seasons noted protection ranging from 54% to 67% during days 0 through 180 postvaccination (*20*). A third multiseason analysis (2011–12 through 2014–15) conducted in Europe noted a decline in VE to 0% at 111 days postvaccination for A(H3N2) viruses. VE against B viruses declined more slowly and VE against A(H1N1) viruses remained roughly stable at 50-55% through the influenza season (*21*).

Variable data concerning presence and rate of waning immunity following influenza vaccination, coupled with the unpredictable timing of the influenza season each year, prevent determination of an optimal time to vaccinate. Programmatic issues are also a consideration. Although some available data indicate that early vaccination (e.g., in July and August) might be associated with suboptimal immunity before the end of the influenza season, particularly among older adults, the relative contribution of potential waning of immunity compared with those of other determinants of the impact of vaccination (e.g., timing and severity of the influenza season, the impact of missed opportunities when individuals delay vaccination and fail to return later in the season, and programmatic constraints) is unknown. Although delaying vaccination might result in greater immunity later in the season, deferral also might result in missed opportunities to vaccinate, as well as difficulties in vaccinating a population within a more constrained time period. Efforts should be structured to optimize vaccination coverage before influenza activity in the community begins.

Vaccination efforts should continue throughout the season because the duration of the influenza season varies, and influenza activity might not occur in certain communities until February or March. Providers should offer influenza vaccine routinely, and organized vaccination campaigns should continue throughout the influenza season, including after influenza activity has begun in the community. Although vaccination by the end of October is recommended, vaccine administered in December or later, even if influenza activity has already begun, is likely to be beneficial in the majority of influenza seasons.

### Guidance for Use in Specific Populations and Situations

#### Populations at Higher Risk for Medical Complications Attributable to Severe Influenza

All persons aged ≥6 months without contraindications should be vaccinated annually. However, vaccination to prevent influenza is particularly important for persons who are at increased risk for severe complications from influenza and for influenza-related outpatient, emergency department, or hospital visits. When vaccine supply is limited, vaccination efforts should focus on delivering vaccination to persons at higher risk for medical complications attributable to severe influenza who do not have contraindications. These persons include (no hierarchy is implied by order of listing):

- All children aged 6 through 59 months;
- All persons aged ≥50 years;
- Adults and children who have chronic pulmonary (including asthma) or cardiovascular (excluding isolated hypertension), renal, hepatic, neurologic, hematologic, or metabolic disorders (including diabetes mellitus);
- Persons who are immunocompromised due to any cause (including immunosuppression caused by medications or by HIV infection);
- Women who are or will be pregnant during the influenza season;
- Children and adolescents (aged 6 months through 18 years) who are receiving aspirin- or salicylate-containing medications and who might be at risk for experiencing Reye syndrome after influenza virus infection;
- Residents of nursing homes and other long-term care facilities;
- American Indians/Alaska Natives; and
- Persons who are extremely obese (body mass index ≥40).

An age-appropriate IIV or RIV4 is suitable for persons in all risk groups. LAIV4 is not recommended for some populations, including some groups listed above. Contraindications and precautions to the use of LAIV4 are noted (Table 2).

#### Persons Who Live with or Care for Persons at Higher Risk for Influenza-Related Complications

All persons aged ≥6 months without contraindications should be vaccinated annually; however, continued emphasis should be placed on vaccination of persons who live with or care for persons at higher risk for influenza-related complications. When vaccine supply is limited, vaccination efforts should focus on delivering vaccination to persons at higher risk for influenza-related complications, as well as persons who live with or care for such persons:

- Health care personnel, including physicians, nurses, and other workers in inpatient and outpatient-care settings, medical emergency-response workers (e.g., paramedics and emergency medical technicians), employees of nursing home and long-term care facilities who have contact with patients or residents, and students in these professions who will have contact with patients. ACIP guidance for immunization of health care personnel has been published previously (*23*);
- Household contacts (including children) and caregivers of children aged ≤59 months (i.e., aged <5 years) and adults aged ≥50 years, particularly contacts of children aged <6 months; and
- Household contacts (including children) and caregivers of persons with medical conditions that put them at higher risk for severe complications from influenza.

Health care personnel and persons who are contacts of persons in these groups (with the exception of those of severely immunocompromised persons who require a protected environment) may receive any influenza vaccine that is otherwise indicated. Persons who care for severely immunocompromised persons requiring a protected environment should receive either IIV or RIV4. ACIP and the Healthcare Infection Control Practices Advisory Committee (HICPAC) have previously recommended that health care personnel who receive LAIV should avoid providing care for severely immunosuppressed patients requiring a protected environment for 7 days after vaccination, and that hospital visitors who have received LAIV should avoid contact with such persons for 7 days after vaccination (*24*). However, such persons need not be restricted from visiting less severely immunosuppressed patients.

#### Children Aged 6 Months Through 8 Years

**Dose volume for children aged 6 through 35 months:** Children aged 6 through 35 months may receive one of three IIV4 products at the appropriate volume for each dose needed. The appropriate dose volume varies by product:

- 0.5 mL FluLaval Quadrivalent (containing 15 *µ*g of HA per vaccine virus),
- 0.5 mL Fluarix Quadrivalent (containing 15 *µ*g of HA per vaccine virus), or
- 0.25 mL Fluzone Quadrivalent (containing 7.5 *µ*g of HA per vaccine virus).

Alternatively, healthy children aged ≥2 years may receive LAIV4, 0.2mL intranasally (0.1 mL each nostril) (see Contraindications and Precautions for the Use of LAIV4; Table 2).

Care should be taken to administer the appropriate volume for each needed dose of the respective product. In each instance the needed volume may be administered from a prefilled syringe containing the appropriate volume (as supplied by the manufacturer), a single dose vial, or a multidose vial. However, if a 0.5 mL single-dose vial of Fluzone Quadrivalent is used for a child aged 6 through 35 months, only half the volume should be administered and the other half should be discarded.

Before November 2016, the only IIV formulations licensed for children aged 6 through 35 months were the 0.25 mL dose formulations of Fluzone and Fluzone Quadrivalent (containing 7.5 *µ*g of HA per vaccine virus). The recommendation for use of a reduced dose volume for children in this age group (half that recommended for persons aged ≥3 years) was based on increased reactogenicity noted among children (particularly younger children) following influenza vaccines, primarily observed with whole-virus inactivated vaccines (*25*–*29*). Vaccines more similar to currently available split-virus inactivated products demonstrated less reactogenicity (*29*). In a randomized trial comparing immunogenicity and safety of 0.5 mL FluLaval Quadrivalent with 0.25 mL Fluzone Quadrivalent, safety and reactogenicity were similar between the two vaccines (*30*). In a post hoc analysis, superior immunogenicity was noted for the B components of FluLaval Quadrivalent among infants aged 6 through 17 months and for unprimed children (those who had not previously received at least 2 doses of influenza vaccine) aged 6 through 35 months.

**Number of doses for children aged 6 months through 8 years:** Evidence from several studies indicates that children aged 6 months through 8 years require 2 doses of influenza vaccine administered a minimum of 4 weeks apart during their first season of vaccination for optimal protection (*31*–*34*). Children aged 6 months through 8 years who have previously received ≥2 total doses of trivalent or quadrivalent influenza vaccine at least 4 weeks apart before July 1, 2018, require only one dose for 2018–19. The 2 doses of influenza vaccine do not have to have been administered in the same season or consecutive seasons. Children in this age group who have not previously received ≥2 doses of trivalent or quadrivalent influenza vaccine before July 1, 2018 require 2 doses for the 2018–19 season. The interval between the 2 doses should be at least 4 weeks (Figure).

**FIGURE:**
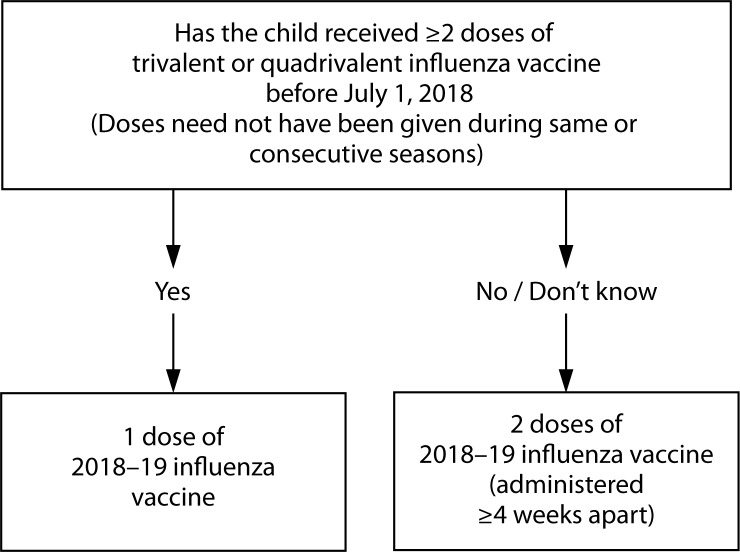
Influenza vaccine dosing algorithm for children aged 6 months through 8 years — Advisory Committee on Immunization Practices, United States, 2018–19 influenza season

#### Pregnant Women

Pregnant and postpartum women have been observed to be at higher risk for severe illness and complications from influenza, particularly during the second and third trimesters. ACIP and the American College of Obstetricians and Gynecologists (*35*–*37*) recommend that all women who are pregnant or who might be pregnant during the influenza season receive influenza vaccine. Any licensed, recommended, and age-appropriate IIV or RIV4 may be used. LAIV4 should not be used during pregnancy. Influenza vaccine can be administered at any time during pregnancy, before and during the influenza season.

Although there is substantial experience with the use of IIVs during pregnancy, data specifically reflecting administration of influenza vaccines during the first trimester are relatively limited (see Safety of Influenza Vaccines—Pregnant Women and Neonates in the Background Document). Most studies have not noted an association between influenza vaccination and adverse pregnancy outcomes. One recent observational Vaccine Safety Datalink (VSD) study conducted during the 2010–11 and 2011–12 seasons noted an association between receipt of IIV containing H1N1pdm09 and risk for spontaneous abortion (miscarriage) in the 28 days after IIV, when an H1N1pdm-09-containing vaccine had also been received the previous season (*38*). This was an unexpected finding, as prior studies (*39*–*47*), and systematic reviews (*48*,*49*), did not find an association between IIV and spontaneous abortion. A follow-up VSD study is in progress (*50*).

There is substantially less experience with more recently licensed IIV products (e.g., quadrivalent, cell culture-based, and adjuvanted vaccines) during pregnancy as compared with previously available products. For RIV (available as RIV3 from 2013–14 through 2017–18, and as RIV4 since 2017–18), data are limited to reports of pregnancies occurring incidentally during clinical trials, VAERS reports, and pregnancy registry reports. Pregnancy registries and surveillance studies exist for some products; information may be found in package inserts (*51*–*56*), available at https://www.fda.gov/BiologicsBloodVaccines/Vaccines/ApprovedProducts/ucm094045.htm for trivalent vaccines and https://www.fda.gov/BiologicsBloodVaccines/Vaccines/ApprovedProducts/ucm295057.htm for quadrivalent vaccines.

#### Older Adults

Because of the vulnerability of this population to severe influenza illness, hospitalization, and death, efficacy and effectiveness of influenza vaccines among older adults is an area of active research (see Immunogenicity, Efficacy, and Effectiveness of Influenza Vaccines: HD-IIV3, aIIV3, and RIV4 for Older Adults in the Background Document). Recent comparative studies of vaccine efficacy/effectiveness against laboratory-confirmed influenza outcomes among older adults have focused on HD-IIV3 (Fluzone High-Dose) (*57*,*58*), RIV4 (Flublok Quadrivalent) (*59*), and aIIV3 (Fluad) (*60*) (see Table in Background Document; https://www.cdc.gov/flu/professionals/acip/2018-19/table3.html). Of note, each of these three vaccines has been studied in comparison to a standard dose, unadjuvanted IIV (SD-IIV3 as the comparator for HD-IIV3 and aIIV3; SD-IIV4 as the comparator for RIV4). To date, HD-IIV3 has been the most extensively studied in this regard, and evidence has accumulated for its superior efficacy and effectiveness compared with SD-IIV3 in this population. Data from studies comparing the efficacy or effectiveness of HD-IIV3, aIIV3, and RIV4 with one another against laboratory-confirmed influenza outcomes among older adults are limited, which prevents recommending one of these three vaccines over another for this population. In comparative safety studies, some injection site and systemic reactions were observed more frequently in older persons vaccinated with HD-IIV3 and aIIV3, compared with unadjuvanted SD-IIV3 (*61*,*62*).

Fluzone High-Dose (HD-IIV3) met prespecified criteria for superior efficacy against laboratory-confirmed influenza to that of SD-IIV3 in a randomized trial conducted over two seasons among 31,989 persons aged ≥65 years, and might provide better protection than SD-IIV3 for this age group (*57*,*63*,*64*). For the primary outcome (prevention of laboratory-confirmed influenza caused by any viral type or subtype and associated with protocol-defined ILI), relative efficacy of HD-IIV3 compared with SD-IIV3 was 24.2% (95% CI = 9.7–36.5%). These findings are further supported by results from retrospective studies of Centers for Medicare and Medicaid Services (CMS) and Veterans Administration data, as well as a cluster-randomized trial of HD-IIV3 versus SD-IIV among older adults in nursing homes (*65*–*69*). A meta-analysis reported that HD-IIV3 provided better protection than SD-IIV3 against ILI (relative VE = 19.5%; 95% CI = 8.6–29.0%); all-cause hospitalizations (relative VE = 9.1%; 95% CI = 2.4–15.3); and hospitalizations due to influenza (relative VE = 17.8%; 95% CI = 8.1–26.5), pneumonia (relative VE = 24.3%; 95% CI = 13.9–33.4), and cardiorespiratory events (relative VE = 18.2%; 95% CI = 6.8–28.1) (*70*).

In an exploratory analysis of data from a single-season randomized trial conducted among 8,604 adults aged ≥50 years, Flublok Quadrivalent (RIV4) was more efficacious than SD-IIV4 (*59*,*71*); however, no claim of superiority was approved for the package insert (*71*). For the primary outcome (RT-PCR-confirmed, protocol-defined ILI caused by any influenza virus type or subtype), the relative VE of RIV4 compared with IIV4 was 30% (95% CI = 10–47). When restricted to persons aged ≥65 years, the relative VE of RIV4 was 17% (95% CI = -20–43%).

Fluad (aIIV3) was more effective against laboratory-confirmed influenza than unadjuvanted SD-IIV3 among adults aged ≥65 years (N = 227; 165 of whom received aIIV3 and 62 IIV3) in an analysis from a small single-season observational study (*60*). Relative effectiveness of aIIV3 compared with unadjuvanted IIV3 was 63% (95% CI = 4–86). No data are yet available concerning efficacy of aIIV3 compared with nonadjuvanted IIV3 against laboratory-confirmed influenza outcomes from a randomized trial in this population.

Additional data concerning these vaccines from studies examining immunogenicity and nonlaboratory-confirmed influenza outcomes are discussed in the Background Document. ACIP will continue to review data concerning the efficacy and effectiveness of these vaccines as more information emerges. No preference is expressed for any one vaccine type. Vaccination should not be delayed if a specific product is not readily available. For persons aged ≥65 years, any age-appropriate IIV formulation (standard-dose or high-dose, trivalent or quadrivalent, unadjuvanted or adjuvanted) or RIV4 are acceptable options.

#### Immunocompromised Persons

Immunocompromised states comprise a heterogeneous range of conditions. In many instances, limited data are available regarding the use of influenza vaccines in the setting of specific immunocompromised states. ACIP recommends that LAIV4 not be used for immunocompromised persons (*72*) because of the uncertain but biologically plausible risk for disease attributable to the vaccine virus. In addition to potential safety issues, immune response to live or inactivated vaccines might be blunted in some clinical situations, such as for persons with congenital immune deficiencies, persons receiving cancer chemotherapy, and persons receiving immunosuppressive medications. For this reason, timing of vaccination might be a consideration (e.g., vaccinating during some period either before or after an immunocompromising intervention).

The Infectious Diseases Society of America (IDSA) has published detailed guidance for the selection and timing of vaccines for persons with specific immunocompromising conditions, including congenital immune disorders, stem cell and solid organ transplant, anatomic and functional asplenia, and therapeutic drug-induced immunosuppression, as well as for persons with cochlear implants or other conditions leading to persistent cerebrospinal fluid-oropharyngeal communication (*73*). Given the paucity of safety data for LAIV in most of these populations, and the availability of alternative vaccines, IIV or RIV4 should be used instead of LAIV for these persons. ACIP will continue to review accumulating data on use of influenza vaccines in these contexts.

#### Persons with a History of Guillain-Barré Syndrome Following Influenza Vaccination

A history of Guillain-Barré Syndrome (GBS) within 6 weeks following a previous dose of any type of influenza vaccine is considered a precaution to vaccination (Table 2). Persons who are not at higher risk for severe influenza complications (see Populations at Higher Risk for Medical Complications Attributable to Severe Influenza) and who are known to have experienced GBS within 6 weeks of a previous influenza vaccination generally should not be vaccinated. As an alternative to vaccination, physicians might consider using influenza antiviral chemoprophylaxis for these persons (*74*). However, the benefits of influenza vaccination might outweigh the risks for certain persons who have a history of GBS and who also are at higher risk for severe complications from influenza.

#### Persons with a History of Egg Allergy

As is the case for other vaccines, influenza vaccines contain various different components that might cause allergic and anaphylactic reactions. Not all such reactions are related to egg proteins; however, the possibility of reactions to influenza vaccines in egg-allergic persons might be of concern to these persons and vaccine providers. Currently available influenza vaccines, with the exceptions of RIV4 (Flublok Quadrivalent) and ccIIV4 (Flucelvax Quadrivalent), are prepared by propagation of virus in embryonated eggs. Only RIV4 is considered egg-free. For ccIIV4, ovalbumin is not directly measured. During manufacture of ccIIV4, viruses are propagated in mammalian cells rather than in eggs. However, one of the four viruses provided to the manufacturer is egg-derived; therefore, egg proteins might potentially be introduced at the start of the manufacturing process. Once these viruses are received by the manufacturer, no eggs are used, and dilutions at various steps during the manufacturing process result in a theoretical maximum of 1.7×10^-8 ^*μ*g/0.5 mL dose of total egg protein (Seqirus, unpublished data, 2018).

Severe allergic reactions to vaccines, although rare, can occur at any time, even in the absence of a history of previous allergic reaction. Therefore, all vaccine providers should be familiar with the office emergency plan, and be certified in cardiopulmonary resuscitation (*72*). For persons who report a history of egg allergy, ACIP recommends the following (based upon the recipient’s previous symptoms after exposure to egg):

- Persons with a history of egg allergy who have experienced only urticaria (hives) after exposure to egg should receive influenza vaccine. Any licensed, recommended, and age-appropriate influenza vaccine (i.e., any IIV, RIV4, or LAIV4) that is otherwise appropriate for the recipient’s health status may be used.
- Persons who report having had reactions to egg involving symptoms other than urticaria (hives), such as angioedema, respiratory distress, lightheadedness, or recurrent emesis; or who required epinephrine or another emergency medical intervention, may similarly receive any licensed, recommended, and age-appropriate influenza vaccine (i.e., any IIV, RIV4, or LAIV4) that is otherwise appropriate for their health status. The selected vaccine should be administered in an inpatient or outpatient medical setting (including, but not necessarily limited to, hospitals, clinics, health departments, and physician offices). Vaccine administration should be supervised by a health care provider who is able to recognize and manage severe allergic reactions.
- A previous severe allergic reaction to influenza vaccine, regardless of the component suspected of being responsible for the reaction, is a contraindication to future receipt of the vaccine.

No postvaccination observation period is recommended specifically for egg-allergic persons. However, ACIP recommends that vaccine providers consider observing patients (seated or supine) for 15 minutes following administration of any vaccine to decrease the risk for injury should syncope occur (*72*).

Persons who are able to eat lightly cooked egg (e.g., scrambled egg) without reaction are unlikely to be allergic. Egg-allergic persons might tolerate egg in baked products (e.g., bread or cake). Tolerance to egg-containing foods does not exclude the possibility of egg allergy. Egg allergy can be confirmed by a consistent medical history of adverse reactions to eggs and egg-containing foods, plus skin and/or blood testing for immunoglobulin E directed against egg proteins (*75*).

Occasional cases of anaphylaxis in egg-allergic persons have been reported to VAERS after administration of influenza vaccines (*76*,*77*). ACIP will continue to review available data regarding anaphylaxis cases following influenza vaccines.

#### Vaccination Issues for Travelers

Travelers who want to reduce the risk for influenza infection should consider influenza vaccination, preferably at least 2 weeks before departure. In particular, persons residing in the United States who are at higher risk for complications of influenza and who were not vaccinated with influenza vaccine during the preceding Northern Hemisphere fall or winter should consider receiving influenza vaccine before departure if they plan to travel to the tropics, with organized tourist groups or on cruise ships, or to the Southern Hemisphere during the Southern Hemisphere influenza season (April–September).

Persons at higher risk who received the previous season’s vaccine before travel should consult with their health care provider to discuss the risk for influenza or other travel-related diseases before embarking on travel during the summer. Persons vaccinated before travel should receive the current vaccine the following fall or winter.

In temperate climate regions of the Northern and Southern hemispheres, influenza activity is seasonal, occurring approximately from October through May in the Northern Hemisphere and April through September in the Southern Hemisphere. In the tropics, influenza occurs throughout the year. Travelers can be exposed to influenza when travelling to an area where influenza is circulating, or when traveling as part of large tourist groups (e.g., on cruise ships) that include persons from areas of the world in which influenza viruses are circulating (*78*–*81*).

Influenza vaccine formulated for the Southern Hemisphere might differ in viral composition from the Northern Hemisphere vaccine. For persons traveling to the Southern Hemisphere during the Southern Hemisphere influenza season, receipt of a current U.S.-licensed Southern Hemisphere formulation influenza vaccine prior to departure might be reasonable, but might not be feasible in many instances. With the exception of the Southern Hemisphere formulation of Fluzone Quadrivalent (IIV4), Southern Hemisphere formulation seasonal influenza vaccines are not licensed in the U.S., and Southern Hemisphere formulations generally are not commercially available in the United States. More information on influenza vaccines and travel is available at https://www.cdc.gov/flu/travelers/travelersfacts.htm.

#### Use of Influenza Antiviral Medications

Administration of IIV or RIV4 to persons receiving influenza antiviral medications for treatment or chemoprophylaxis is acceptable. However, influenza antiviral medications may reduce the effectiveness of LAIV4 if given within 48 hours before to 14 days after LAIV4 (*82*). Persons who receive influenza antiviral medications during this period surrounding receipt of LAIV4 may be revaccinated with another appropriate vaccine formulation (e.g., IIV or RIV4).

#### Administration of Influenza Vaccines with Other Vaccines

In general, data regarding simultaneous or sequential administration for the many potential combinations of vaccines are limited. Therefore, following the ACIP General Best Practice Guidelines for Immunization is prudent (*72*). IIVs and RIV4 may be administered concomitantly or sequentially with other inactivated vaccines or with live vaccines. Injectable vaccines that are given concomitantly should be administered at separate anatomical sites. LAIV4 may be administered simultaneously with other live or inactivated vaccines. However, following administration of a live vaccine (such as LAIV4), at least 4 weeks should pass before another live vaccine is administered.

Relatively limited data are available on the concomitant administration of influenza vaccines with other vaccines. In a study comparing the immunogenicity of IIV and live attenuated zoster vaccine given either concomitantly or separated by a 4-week interval to adults aged ≥50 years, antibody responses were similar for both schedules (*83*). In some studies, reduced responses have been noted to PCV13 (*84*,*85*), tetanus antigens (*86*), and pertussis antigens (*86*) when co-administered with IIV; in most instances the clinical significance of this is uncertain. Reassuring safety profiles have been noted for simultaneous administration of IIV with live attenuated zoster vaccine (*83*), PCV13 (*84*,*85*), PPSV23 (*87*) and Tdap (*86*) among adults and of Tdap among pregnant women (*88*). Increased prevalence of injection site and/or systemic adverse reactions has been noted with concurrent administration in some of these studies, but these symptoms have generally been reported to be mild or moderate.

Among children, co-administration of IIV and PCV13 was associated with increased risk for fever on the day of vaccination and the day following (i.e., days 0–1 postvaccination) in children aged 6 through 23 months in a study conducted during the 2011–12 season (*89*). Increased risk for febrile seizures in this age group has been noted within days 0–1 following co-administration of IIV with PCV7, PCV13, or DTaP-containing vaccines during the 2006–07 through 2010–11 seasons (*90*), and with PCV13 during the 2014–15 season (*91*). While frightening to parents, most febrile seizures are brief and have a good prognosis (*92*). After considering risks and benefits, no changes in the recommendations for administration of these vaccines were made, and these vaccines may be given concomitantly. Surveillance of febrile seizures is ongoing through VAERS, and the VSD annual influenza vaccine safety surveillance includes monitoring for seizures following vaccination.

Studies of concomitant administration of LAIV with other vaccines are limited. Concurrent administration to children of LAIV3 with MMR and varicella vaccine was not associated with diminished immunogenicity to antigens in any of the vaccines in one study (*93*); diminished response to rubella was observed in another study examining co-administration of LAIV3 and MMR (*94*). No safety concerns were revealed in these studies.

In recent years several vaccines containing novel, nonaluminum adjuvants have been licensed for use in the United States. These include AS01_B_ (in Shingrix, recombinant zoster subunit vaccine); MF59 (in Fluad, aIIV3); and cytosine phosphoguanine oligodeoxynucleotide (in Heplisav-B, recombinant hepatitis B surface antigen vaccine). Data are limited regarding co-administration of these vaccines with other adjuvanted or unadjuvanted vaccines. Co-administration of Shingrix with unadjuvanted IIV4 has been studied; no evidence of decreased immunogenicity or safety concerns were noted (*95*). The immunogenicity and safety of simultaneous or sequential administration of two novel adjuvant-containing vaccines has not been evaluated, and the ideal interval between such vaccines when given sequentially is not known. In the study of Shingrix and IIV4 discussed above, most reactogenicity symptoms resolved within 4 days. Given unknown but theoretical concerns of increased reactogenicity when administering two novel adjuvant-containing vaccines, and the availability of nonadjuvanted influenza vaccine options, selection of a nonadjuvanted influenza vaccine may be considered in situations where influenza vaccine and another vaccine containing a novel adjuvant are to be administered concomitantly. However, vaccination should not be delayed if a specific product is not available. Vaccines with newer adjuvants, like other vaccines, should be administered at separate sites from other vaccines that are given concomitantly (*72*).

## Influenza Vaccine Composition and Available Products

### Influenza Vaccine Composition for the 2018–19 Season

All influenza vaccines licensed in the United States will contain components derived from influenza viruses antigenically similar to those recommended by FDA (https://www.fda.gov/downloads/AdvisoryCommittees/CommitteesMeetingMaterials/BloodVaccinesandOtherBiologics/VaccinesandRelatedBiologicalProductsAdvisoryCommittee/UCM602610.pdf). Both trivalent and quadrivalent influenza vaccines will be available in the United States. The 2018–19 U.S. influenza vaccines will contain hemagglutinin derived from the following:

- an A/Michigan/45/2015 (H1N1)pdm09–like virus,
- an A/Singapore/INFIMH-16-0019/2016 (H3N2)–like virus, and
- a B/Colorado/06/2017–like virus (Victoria lineage).

The 2018–19 U.S. quadrivalent vaccines will contain the same three antigens and an additional influenza B virus component, a B/Phuket/3073/2013–like virus (Yamagata lineage). Compared with the 2017–18 season, the composition for 2018–19 represents changes in the A(H3N2) and B (Victoria) components of both the trivalent and quadrivalent vaccines.

### Vaccine Products for the 2018–19 Season

A variety of influenza vaccine products are licensed and available from several different manufacturers (Table 1). For many vaccine recipients, more than one type or brand of vaccine might be appropriate within approved indications and ACIP recommendations. A licensed, age-appropriate influenza vaccine product should be used. Not all products are likely to be uniformly available in any practice setting or locality. Vaccination should not be delayed in order to obtain a specific product when an appropriate one is already available. Within these guidelines and approved indications, where more than one type of vaccine is appropriate and available, no preferential recommendation is made for use of any influenza vaccine product over another.

Since the publication of the previous season’s guidelines, there have been changes in the licensed age indications for Afluria Quadrivalent and Fluarix Quadrivalent. Further details are in the Recent Influenza Vaccine Product Approvals section. New licensures and changes to FDA-approved labeling might occur subsequent to publication of this report. As these changes occur, they will be reflected in the online version of Table 1, available at https://www.cdc.gov/flu/protect/vaccine/vaccines.htm.

### Dosage, Administration, Contraindications, and Precautions

#### Inactivated Influenza Vaccines (IIVs)

**Available products:** IIVs comprise multiple products (Table 1). Both quadrivalent and trivalent formulations are available. For the 2018–19 season, it is anticipated that all standard-dose, unadjuvanted inactivated influenza vaccines will be quadrivalent (IIV4s), with the exception of Afluria, which will be available in both trivalent (IIV3) and quadrivalent formulations. High-dose unadjuvanted inactivated influenza vaccine (HD-IIV3, Fluzone High-Dose) and adjuvanted inactivated influenza vaccine (aIIV3, Fluad) will be trivalent.

With one exception, U.S.-licensed IIVs are manufactured through propagation of virus in eggs. The exception, the cell culture-based vaccine Flucelvax Quadrivalent (ccIIV4), contains vaccine viruses propagated in Madin-Darby canine kidney cells. Flucelvax Quadrivalent is not considered egg-free, as one of the initial vaccine viruses provided to the manufacturer is egg-derived. For the 2018–19 season, the influenza A (H3N2) and both influenza B components will be cell-derived; the influenza A (H1N1) component will be egg-derived.

With one exception, IIVs licensed in the United States contain no adjuvant. The exception, Fluad (aIIV3) contains the adjuvant MF59.

There are IIVs that are licensed for persons as young as 6 months of age. However, age indications for the various individual IIVs differ (Table 1). Only age-appropriate products should be administered. Providers should consult package inserts and updated CDC/ACIP guidance for current information.

**Dosage and administration:** All IIV preparations available for the 2018–19 season contain 15 *µ*g of HA per vaccine virus strain (45 *µ*g total for IIV3s and 60 *µ*g total for IIV4s) per 0.5 mL dose, with the exception of Fluzone High-Dose. Fluzone High-Dose (HD-IIV3), an IIV3 licensed for persons aged ≥65 years, contains 60 *µ*g of HA per vaccine virus strain (180 *µ*g total) (*63*).

For children aged 6 through 35 months, three IIV4 products are licensed by FDA. The approved dose volumes differ for these products. For each recommended dose, children in this age group may receive either 1) 0.5 mL of FluLaval Quadrivalent (*51*), which contains 15 *µ*g of HA per virus; 2) 0.5mL of Fluarix Quadrivalent (*52*), which contains 15 *µ*g of HA per virus; or 3) 0.25 mL of Fluzone Quadrivalent (*56*), which contains 7.5 *µ*g of HA per virus. Care must be taken to administer each at the appropriate dose for each product in this age group. If prefilled syringes are not available, the dose can be taken from a single-use or multidose vial, at the appropriate volume for the given product.

Children aged 36 months through 17 years and adults aged ≥18 years who are receiving IIV should receive 0.5 mL per dose. If a smaller intramuscular vaccine dose (e.g., 0.25 mL) is administered inadvertently to a person aged ≥36 months, an additional 0.25 mL dose should be administered to provide a full dose (0.5 mL). If the error is discovered later (after the patient has left the vaccination setting), a full 0.5 mL dose should be administered as soon as the patient can return. Vaccination with a formulation approved for adult use should be counted as a dose if inadvertently administered to a child.

IIVs are administered intramuscularly. For adults and older children, the deltoid is the preferred site. Infants and younger children should be vaccinated in the anterolateral thigh. Additional specific guidance regarding site selection and needle length for intramuscular administration is provided in the ACIP General Best Practice Guidelines for Immunization (*72*). Two IIVs, Afluria (IIV3) and Afluria Quadrivalent (IIV4), are licensed for intramuscular administration via jet injector (Stratis, Pharmajet) for persons aged 18 through 64 years (*53*,*96*).

**Trivalent versus quadrivalent IIVs:** Both trivalent and quadrivalent IIVs will be available during the 2018–19 season. Quadrivalent vaccines contain one virus from each of the two influenza B lineages (one B/Victoria virus and one B/Yamagata virus), whereas trivalent vaccines contain one influenza B virus from one lineage. Quadrivalent vaccines are thus designed to provide broader protection against circulating influenza B viruses. However, no preference is expressed for either IIV3 or IIV4.

**Contraindications and precautions for the use of IIVs:** Manufacturer package inserts and updated CDC/ACIP guidance should be consulted for current information on contraindications and precautions for individual vaccine products. In general, history of severe allergic reaction to the vaccine or any of its components (including egg) is a labeled contraindication to the receipt of IIVs (Table 2). However, ACIP makes specific recommendations for the use of influenza vaccine for persons with egg allergy (see Persons with a History of Egg Allergy). Influenza vaccine is not recommended for persons with a history of severe allergic reaction to the vaccine or to components other than egg. Information about vaccine components is located in package inserts from each manufacturer. Prophylactic use of antiviral agents is an option for preventing influenza among persons who cannot receive vaccine (*74*).

Moderate or severe acute illness with or without fever is a general precaution for vaccination (*72*). GBS within 6 weeks following a previous dose of influenza vaccine is considered a precaution for use of influenza vaccines (Table 2).

#### Recombinant Influenza Vaccine (RIV4)

**Available products:** One RIV product, Flublok Quadrivalent (RIV4), is expected to be available for the 2018–19 influenza season. RIV4 is indicated for persons aged ≥18 years. RIV4 is manufactured without the use of influenza viruses; therefore, similarly to IIVs, no shedding of vaccine virus will occur. RIV4 is produced without the use of eggs, and is egg-free. No preference is expressed for RIV4 versus IIVs within specified indications.

**Dosage and administration:** RIV4 is administered by intramuscular injection. A 0.5 mL dose contains 45 µg of HA derived from each vaccine virus (180 *µ*g total) (*55*).

**Contraindications and precautions for the use of RIV4:** RIV4 is contraindicated in persons who have had a severe allergic reaction to any component of the vaccine. Moderate or severe acute illness with or without fever is a general precaution for vaccination (*72*). GBS within 6 weeks following a previous dose of influenza vaccine is considered a precaution for use of influenza vaccines (Table 2). RIV4 is not licensed for use in children aged <18 years.

#### Live Attenuated Influenza Vaccine (LAIV4)

**Available products:** One LAIV4 product, FluMist Quadrivalent, is expected to be available during the 2018–19 influenza season. LAIV4 is licensed for persons aged 2 through 49 years.

**Dosage and administration:** LAIV4 is administered intranasally using the supplied prefilled, single-use sprayer containing 0.2 mL of vaccine. Approximately 0.1 mL (i.e., half of the total sprayer contents) is sprayed into the first nostril while the recipient is in the upright position. An attached dose-divider clip is removed from the sprayer to administer the second half of the dose into the other nostril. If the vaccine recipient sneezes immediately after administration, the dose should not be repeated. However, if nasal congestion is present that might impede delivery of the vaccine to the nasopharyngeal mucosa, deferral of administration should be considered until resolution of the illness, or another appropriate vaccine should be administered instead.

**Contraindications and precautions for the use of LAIV4:** Per the package insert, LAIV4 is contraindicated for persons with a history of severe allergic reaction to any component of the vaccine or to a previous dose of any influenza vaccine, and in children and adolescents receiving concomitant aspirin or salicylate-containing medications (Table 2). While LAIV is an egg-based vaccine, ACIP makes specific recommendations for the use of influenza vaccine for persons with egg allergy (see Persons with a History of Egg Allergy). In addition to the labeled contraindications (other than allergy to egg), ACIP also recommends that LAIV should not be administered to the following groups:

- Children aged 2 through 4 years who have received a diagnosis of asthma or whose parents or caregivers report that a health care provider has told them during the preceding 12 months that their child had wheezing or asthma or whose medical record indicates a wheezing episode has occurred during the preceding 12 months;
- Persons who are immunocompromised due to any cause (including immunosuppression caused by medications and HIV infection);
- Close contacts and caregivers of severely immunosuppressed persons who require a protected environment;
- Pregnant women; and
- Persons who have received influenza antiviral medications within the previous 48 hours.

Moderate or severe acute illness with or without fever is a general precaution for vaccination (*72*). GBS within 6 weeks following a previous dose of influenza vaccine is considered a precaution for use of influenza vaccines. Additional precautions specific to LAIV4 include asthma in persons aged ≥5 years and presence of an underlying medical condition (other than the instances listed in which LAIV4 should not be used) that might predispose to complications after wild-type influenza infection (see Populations at Higher Risk for Medical Complications Attributable to Severe Influenza; Table 2).

### Recent Influenza Vaccine Product Approvals

Since the publication of the previous season’s guidance, there have been two labeling changes for previously licensed influenza vaccines. These include expansions in the age indications for Afluria Quadrivalent and Fluarix Quadrivalent.

#### Afluria Quadrivalent (IIV4)

In August 2017, FDA approved expansion of the licensed age indication for Afluria Quadrivalent. Previously licensed for persons aged ≥18 years, Afluria Quadrivalent is now licensed for persons aged ≥5 years. In a multicenter observer-blind trial in which 2,278 children aged 5 through 17 years were randomized 3:1 to receive Afluria Quadrivalent or a licensed comparator IIV4, Afluria Quadrivalent met criteria for noninferior immunogenicity for all four vaccine virus strains (*97*). The overall safety profiles were comparable. Fever occurred more frequently among Afluria Quadrivalent recipients, but the difference was not statistically significant. Myalgia was more frequent among Afluria Quadrivalent recipients aged 9 through 17 years, (relative risk: 1.50; 95% CI = 1.03–2.19) but in most instances was mild to moderate in severity.

#### Fluarix Quadrivalent (IIV4)

In January 2018, FDA approved expansion of the licensed age indication for Fluarix Quadrivalent (IIV4). Previously licensed for persons aged ≥3 years, Fluarix Quadrivalent is now licensed for persons aged ≥6 months. The approved dose volume is 0.5 mL for all ages (*52*,*98*).

In a multicenter randomized observer-blind controlled trial conducted among 12,018 children aged 6–35-months in 13 countries that compared the efficacy, immunogenicity, and safety of 0.5 mL Fluarix Quadrivalent with noninfluenza control vaccines (pneumococcal conjugate vaccine, hepatitis A vaccine, or varicella vaccine, depending upon participant age and prior influenza receipt status), vaccine efficacy (VE) of Fluarix Quadrivalent for reverse transcriptase polymerase chain reaction (RT-PCR)-confirmed influenza infection of any severity due to any virus was 50% (97.5% CI = 42–57). VE against moderate-to-severe RT-PCR-confirmed influenza was 63% (97.5% CI = 52–72). Moderate to severe influenza was defined as RT-PCR-confirmed influenza with any of the following: fever >39.0^°^C; physician-diagnosed acute otitis media, lower respiratory infection, or serious extrapulmonary complication of influenza; intensive care unit hospitalization; or requirement for supplemental oxygen for >8 hours. Considering culture-confirmed influenza due to viruses matching those in the vaccine, VE against influenza of any severity was 60% (95% CI = 49–69) and against moderate to severe influenza was 78% (95% CI = 64–87). Prevalence of injection site and systemic solicited reactions, unsolicited adverse events, and medically attended adverse events were generally similar between the treatment arms (*98*,*99*).

### Storage and Handling of Influenza Vaccines

In all instances, approved manufacturer packaging information should be consulted for authoritative guidance concerning storage and handling of all influenza vaccines. Vaccines should be protected from light and stored at recommended temperatures. In general, influenza vaccines are recommended to be stored refrigerated between 2° to 8°C (36^°^ to 46^°^F) and should not be frozen. Vaccine that has frozen should be discarded. Single-dose vials should not be accessed for more than one dose. Multiple-dose vials should be returned to recommended storage conditions between uses, and once first accessed should not be kept beyond the recommended period of time. For information on permissible temperature excursions and other departures from recommended storage conditions that are not discussed in the package labelling, contact the manufacturer. Vaccines should not be used after the expiration date on the label.

## Additional Sources for Information Regarding Influenza and Influenza Vaccines

### Influenza Surveillance, Prevention, and Control

Updated information regarding influenza surveillance, detection, prevention, and control is available at https://www.cdc.gov/flu. U.S surveillance data are updated weekly during October–May on FluView (https://www.cdc.gov/flu/weekly). In addition, periodic updates regarding influenza are published in *MMWR* (https://www.cdc.gov/mmwr). Additional information regarding influenza vaccine can be obtained from CDC-INFO by calling 1-800-232-4636. State and local health departments should be consulted about availability of influenza vaccine, access to vaccination programs, information related to state or local influenza activity, reporting of influenza outbreaks and influenza-related pediatric deaths, and advice concerning outbreak control.

### Vaccine Adverse Event Reporting System

The National Childhood Vaccine Injury Act of 1986 requires health care providers to report any adverse event listed by the vaccine manufacturer as a contraindication to further doses of the vaccine, or any adverse event listed in the VAERS Table of Reportable Events Following Vaccination (https://vaers.hhs.gov/docs/VAERS_Table_of_Reportable_Events_Following_Vaccination.pdf) that occurs within the specified time period after vaccination. In addition to mandated reporting, health care providers are encouraged to report any clinically significant adverse event following vaccination to VAERS. Information on how to report a vaccine adverse event is available at https://vaers.hhs.gov/index.html. Additional information on VAERS and vaccine safety is available by emailing info@vaers.org or by calling 1-800-822-7967.

### National Vaccine Injury Compensation Program

The National Vaccine Injury Compensation Program (VICP), established by the National Childhood Vaccine Injury Act of 1986, as amended, provides a mechanism through which compensation can be paid on behalf of a person determined to have been injured or to have died as a result of receiving a vaccine covered by VICP. The Vaccine Injury Table (https://www.hrsa.gov/vaccinecompensation/vaccineinjurytable.pdf) lists the vaccines covered by VICP and the associated injuries and conditions (including death) that might receive a legal presumption of causation. If the injury or condition is not on the Table, or does not occur within the specified time period on the Table, persons must prove that the vaccine caused the injury or condition. Eligibility for compensation is not affected by whether a covered vaccine is used off-label or inconsistently with recommendations.

To be eligible for compensation under VICP, a claim must be filed within 3 years after the first symptom of the vaccine injury. Death claims must be filed within 2 years of the vaccine-related death and not more than 4 years after the start of the first symptom of the vaccine-related injury from which the death occurred. When a new vaccine is covered by VICP or when a new injury/condition is added to the Table, claims that do not meet the general filing guidelines must be filed within 2 years from the date the vaccine or injury/condition is added to the Table for injuries or deaths that occurred up to 8 years before the Table change (*100*). Persons of all ages who receive a VICP-covered vaccine might be eligible to file a claim. Additional information is available at https://www.hrsa.gov/vaccinecompensation or by calling 1-800-338-2382.

### Additional Resources

#### ACIP Statements

- General Best Practice Guidelines for Immunization: Best Practices Guidance of the Advisory Committee on Immunization Practices (ACIP). https://www.cdc.gov/vaccines/hcp/acip-recs/general-recs/index.html
- Immunization of Healthcare Personnel: Recommendations of the Advisory Committee on Immunization Practices (ACIP), 2011. MMWR Recomm Rep 2011;60(No. RR-7). https://www.cdc.gov/mmwr/preview/mmwrhtml/rr6007a1.htm
- Recommended Immunization Schedules for Adults Aged 19 Years or Older, United States, 2018. https://www.cdc.gov/vaccines/schedules/hcp/adult.html
- Recommended Immunization Schedule for Children and Adolescents Aged 18 years or Younger, United States, 2018. https://www.cdc.gov/vaccines/schedules/hcp/child-adolescent.html

#### Vaccine Information Sheets (VISs)

- VIS for IIV and RIV: https://www.cdc.gov/vaccines/hcp/vis/vis-statements/flu.pdf
- VIS for LAIV: https://www.cdc.gov/vaccines/hcp/vis/vis-statements/flulive.pdf

#### Influenza Vaccine Package Inserts

- Trivalent Vaccines: https://www.fda.gov/BiologicsBloodVaccines/Vaccines/ApprovedProducts/ucm094045.htm
- Quadrivalent Vaccines: https://www.fda.gov/BiologicsBloodVaccines/Vaccines/ApprovedProducts/ucm295057.htm

#### CDC Influenza Antiviral Guidance

- Antiviral Drugs: Information for Healthcare Professionals: https://www.cdc.gov/flu/professionals/antivirals/index.htm

#### American Academy of Pediatrics (AAP) Guidance

- AAP Recommendations for Prevention and Control of Influenza in Children (Red Book Online): https://redbook.solutions.aap.org/ss/influenza-resources.aspx

#### Infectious Diseases Society of America (IDSA) Guidance

- 2013 IDSA Clinical Practice Guideline for Vaccination of the Immunocompromised Host: https://academic.oup.com/cid/article/58/3/e44/336537

#### American College of Obstetricians and Gynecologists (ACOG)

- Influenza Vaccination During Pregnancy, ACOG Committee Opinion No. 732: https://www.acog.org/Clinical-Guidance-and-Publications/Committee-Opinions/Committee-on-Obstetric-Practice/Influenza-Vaccination-During-Pregnancy*A list of Work Group members may be found on page 20 of this report. A Disclosure of Relationships may be found on page 19.
